# Supplementary figures and images for: Analysis of AlphaFold and molecular dynamics structure predictions of mutations in serpins
Source: PLoS One. 2024 Jul 5;19(7):e0304451. doi: 10.1371/journal.pone.0304451 (PMC11226102; doi:10.1371/journal.pone.0304451)

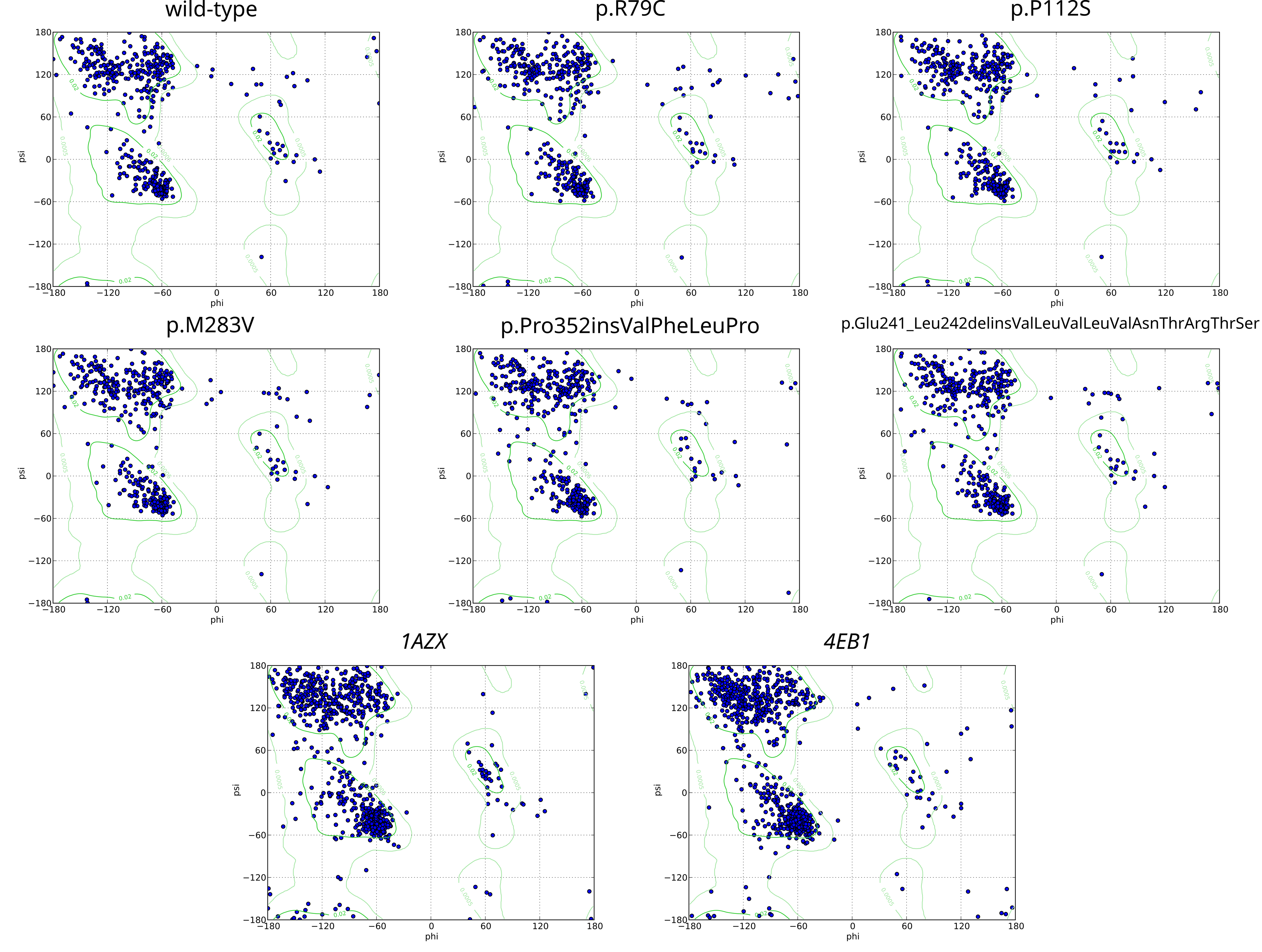

Supplement: S1 Fig — Mutant structures correspond to AlphaFold prediction for said variant. Plots entitled in caps and italics refer to stated PDB crystal structures. (TIF) [file pone.0304451.s001.tif]

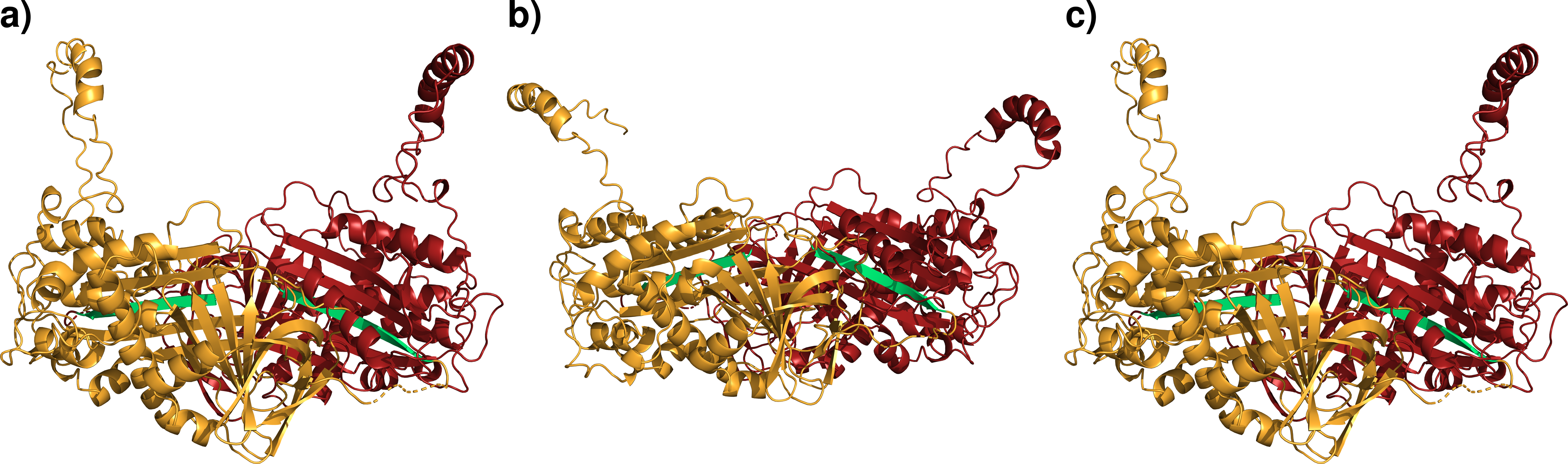

Supplement: S2 Fig — a) M2. b) M4. c) Wild-type. Green and blue: antithrombin. Purple: exchanged β-strand. (TIF) [file pone.0304451.s002.tif]

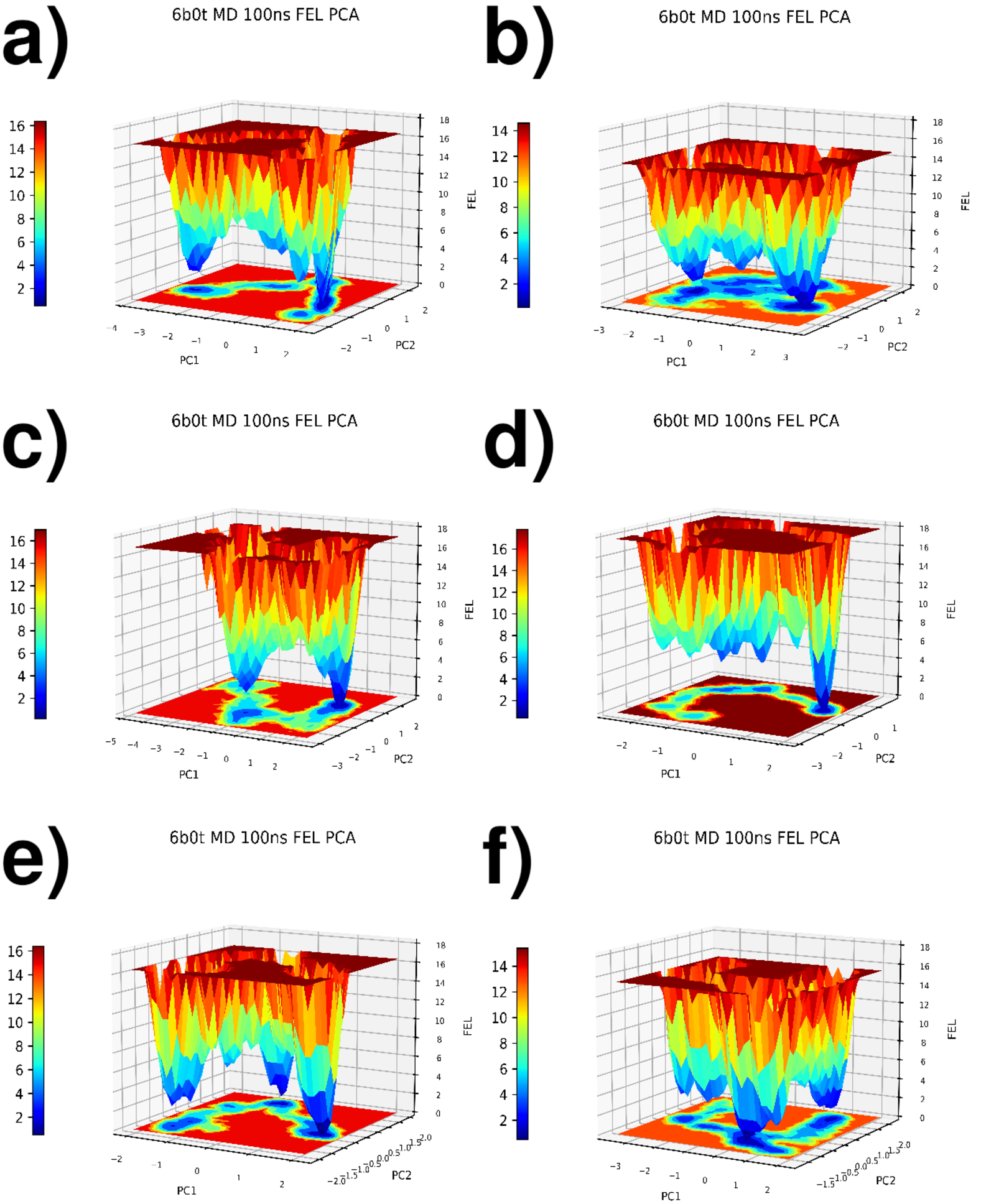

Supplement: S3 Fig — The X and Y axes represent PC1 and PC2 PCA components and the Z axis free energy value. a) Wild-type, b) M1, c) M2, d) M3, e) M4, f) M5. (TIF) [file pone.0304451.s003.tif]
