# Supplementary material for: Analysis of AlphaFold and molecular dynamics structure predictions of mutations in serpins
Source: PLoS One. 2024 Jul 5;19(7):e0304451. doi: 10.1371/journal.pone.0304451 (PMC11226102; doi:10.1371/journal.pone.0304451)
Supplement: S1 Table — (PDF) [file pone.0304451.s005.pdf]

Variant	Origin	Prediction	Score
p.Arg79Cys	ACMG	Pathogenic	-
	MetaLR	Damaging	0.6737
	MetaSVM	Damaging	0.4294
	MetaRNN	Damaging	0.8396
	REVEL	Pathogenic	0.7429
	BayesDel addAF	Damaging	0.1701
	BayesDelnoAF	Damaging	0.2987
	DEOGEN2	Damaging	0.8176
	EIGEN	Pathogenic	0.6868
	EIGEN PC	Pathogenic	0.6482
	FATHMM	Damaging	-1.69
	FATHMM-MKL	Damaging	0.9084
	FATHMM-XF	Damaging	0.9012
	LIST-S2	Damaging	0.9565
	LRT	Deleterious	0
	M-CAP	Damaging	0.1211
	MVP	Pathogenic	0.9571
	MutPred	Pathogenic	0.755
	Mutation assessor	Medium	2.19
	MutationTaster	Disease causing	1
	PROVEAN	Damaging	-2.68
	PrimateAI	Tolerated	0.7381
	SIFT	Damaging	0
	SIFT4G	Damaging	0.001, 0.002
p.Pro112Ser	ACMG	Pathogenic	-
	MetaLR	Damaging	0.9576
	MetaSVM	Damaging	10.973
	MetaRNN	Damaging	0.9775
	REVEL	Pathogenic	0.9649
	BayesDel addAF	Damaging	0.5951
	BayesDel noAF	Damaging	0.617
	DEOGEN2	Damaging	0.9388
	EIGEN	Pathogenic	0.9768
	EIGEN PC	Pathogenic	0.9297
	FATHMM	Damaging	-4.28
	FATHMM-MKL	Damaging	0.9387
	FATHMM-XF	Damaging	0.9657
	LIST-S2	Damaging	0.9547, 0.9508
	LRT	Deleterious	0
	M-CAP	Damaging	0.3203
	MVP	Pathogenic	0.9766
	MutPred	Pathogenic	0.932
	Mutation assessor	Medium	3.09
	MutationTaster	Disease causing	1
	PROVEAN	Damaging	-6.64
	PrimateAI	Tolerated	0.7232
	SIFT	Damaging	0
	SIFT4G	Damaging	0, 0.019
p.Met283Val	ACMG	Uncertain significance	-

	MetaLR	Damaging	0.6121
	MetaSVM	Damaging	0.1556
	MetaRNN	Damaging	0.8389
	REVEL	Pathogenic	0.689
	BayesDel addAF	Damaging	0.2569
	BayesDel noAF	Damaging	0.1314
	DEOGEN2	Damaging	0.9168
	EIGEN	Benign	-0.0229
	EIGEN PC	Benign	0.006645
	FATHMM	Damaging	-2.37
	FATHMM-MKL	Damaging	0.9347
	FATHMM-XF	Damaging	0.8248
	LIST-S2	Tolerated	0.7519
	LRT	Deleterious	0
	M-CAP	Damaging	0.07539
	MVP	Pathogenic	0.9613
	MutPred	Pathogenic	0.942
	Mutation assessor	Medium	3.24
	MutationTaster	Disease causing	0.9997
	PROVEAN	Damaging	-2.77
	PrimateAI	Tolerated	0.3828
	SIFT	Damaging	0.019
	SIFT4G	Damaging	0.037
	EVE	Uncertain	0.4142
p.Pro352insValPheLeuPro	ClinVar	Pathogenic	-
p.Glu241_Leu242delinsValLeuValLeuValAsnThrArgThrSer	-	-	-

S2 Table. Pathogenicity predictions for selected variants.
