# Supplementary material for: Analysis of AlphaFold and molecular dynamics structure predictions of mutations in serpins
Source: PLoS One. 2024 Jul 5;19(7):e0304451. doi: 10.1371/journal.pone.0304451 (PMC11226102; doi:10.1371/journal.pone.0304451)
Supplement: S2 Table — An analysis of Chi1 and Chi2 dihedral angles from crystal structures and AlphaFold Predictions. (DOCX) [file pone.0304451.s006.docx]

**S6 Table. Comparing side-chain conformations of the wild-type and M5 mutant.** An analysis of Chi1 and Chi2 dihedral angles from crystal structures and AlphaFold Predictions.

| **Residue** | **1AZX** | | | **4EB1** | | | **M5 AlphaFold** | | |
| --- | --- | --- | --- | --- | --- | --- | --- | --- | --- |
|  | **Χ 1** | **Χ 2** | **G-Factor** | **Χ 1** | **Χ 2** | **G-Factor** | **Χ 1** | **Χ 2** | **G-Factor** |
| **Ser204** | -53.55 | - | -3.66 | 161.58 | - | -4.586 | -57.52 | - | -2.019 |
| **Glu205** | -63.73 | -61.77 | -3.869 | 79.17 | 169.79 | -6.644 | -53.39 | -55.45 | -5.408 |
| **Ala206** | - | - | - | - | - | - | - | - | - |
| **Ile207** | -74.99 | 178.76 | -3.493 | -50.67 | 171.42 | -3.624 | -70.29 | 169.15 | -1.989 |
| **Asn208** | 59.26 | 50.7 | -5.672 | -160.89 | 86-34 | -8.29 | -66.78 | -16.96 | -3.176 |
| **Thr211** | -54.47 | - | -2.658 | -43.57 | - | -4.961 | 33.29 | - | -7.32 |
| **Val212** | -56.61 | - | -2.159 | 177.97 | - | -1.083 | 178.49 | - | -1.083 |
| **Leu213** | -178.1 | 60.9 | -2.426 | -171.95 | 63.81 | -3.693 | -172.75 | 67 | -3.927 |
| **Val214** | 175.97 | - | -1.083 | -172.42 | - | -3.004 | -176.65 | - | -1.083 |
| **Leu215** | -90.1 | 66.32 | -6.393 | -80.22 | 61.68 | -5.544 | -178.74 | 63.01 | -2.426 |
