# Supplementary material for: Analysis of AlphaFold and molecular dynamics structure predictions of mutations in serpins
Source: PLoS One. 2024 Jul 5;19(7):e0304451. doi: 10.1371/journal.pone.0304451 (PMC11226102; doi:10.1371/journal.pone.0304451)
Supplement: S3 Table — A comparison of G-factors from crystal structures and AlphaFold predictions. (DOCX) [file pone.0304451.s007.docx]

**S7 Table. Assessing the quality of the wild-type and M5 mutant structures.** A comparison of G-factors from crystal structures and AlphaFold predictions.

| **Residue** | **1AZX** | | | **4EB1** | | | **M5 AlphaFold** | | |
| --- | --- | --- | --- | --- | --- | --- | --- | --- | --- |
|  | **Backbone** | **Sidechain** | **Total** | **Backbone** | **Sidechain** | **Total** | **Backbone** | **Sidechain** | **Total** |
| **Ser204** | -4.287 | -.366 | -7.946 | -5.91 | -4.586 | -10.496 | -4.503 | -2.019 | -6.522 |
| **Glu205** | -10.52 | -3.869 | -14.389 | -6.697 | -6.644 | -13.341 | disallowed | -5.408 | disallowed |
| **Ala206** | -2.958 | - | -2.958 | -6.326 | - | -6.326 | -2.772 | - | -2.772 |
| **Ile207** | -4.15 | -3.493 | -7.643 | -3.27 | -3.624 | -6.894 | -2.785 | -1.989 | -4.774 |
| **Asn208** | -6.518 | -5.672 | -12.19 | -5.57 | -8.29 | -13.86 | -2.583 | -3.176 | -5.759 |
| **Thr211** | -5.42 | -2.658 | -8.079 | -6.222 | -4.961 | -11.183 | -7.127 | -7.32 | -14.447 |
| **Val212** | -8.445 | -2.159 | -10.604 | -6.36 | -1.083 | -7.443 | -4.407 | -1.083 | -5.489 |
| **Leu213** | -6.027 | -2.426 | -8.453 | -6.076 | -3.693 | -9.678 | -4.606 | -3.927 | -8.533 |
| **Val214** | -4.266 | -1.083 | -5.348 | -3.956 | -3.004 | -6.96 | -3.647 | -1.083 | -4.73 |
| **Leu215** | -4.114 | -6.393 | -10.507 | -4.712 | -5.5544 | -10.256 | -4.665 | -2.426 | -7.091 |
