# Supplementary material for: Analysis of AlphaFold and molecular dynamics structure predictions of mutations in serpins
Source: PLoS One. 2024 Jul 5;19(7):e0304451. doi: 10.1371/journal.pone.0304451 (PMC11226102; doi:10.1371/journal.pone.0304451)
Supplement: Attachment — Submitted filename: review.pdf [file pone.0304451.s009.pdf]

In this manuscript, authors have used Alphafold, Rosetta and short molecular dynamics simulations to predict structural properties of 5 mutants/variants of serpins. As it is already known in the field, the authors find that these tools are not powerful enough to predict the effects of mutations on the protein structure. Thus, this study does not provide any new insights. Besides, the authors have not provided sufficient details on the methods and analysis presented in this manuscript. Therefore, I cannot recommend this manuscript for publication. Please find my specific comments below:

1. Authors describe various conformational states of serpins using the terms “native”, “stressed”, “hyperstable”, and “relaxed structure”. However, these terms are not properly defined in the introduction and there is an ambiguity in the usage of these terms in the manuscript. This makes it difficult to understand which structural state is being discussed. Authors must define these terms and be consistent in the usage of these terms throughout the manuscript. Besides, figures showing these structural states is required for the purpose of clarity.
2. In the methods section, it is mentioned that the data was collected from 350 patients, and 135 unique genetic variations were found among 250 samples. What about remaining 100 patients? Further, the authors mention that anti-FXa and anti-FIIa activities measured. Authors must explain anti-FXa and anti-FIIa activities.
3. What was the reference structure used for computing RMSD values given in Table 2?
4. In Table 3, what is the meaning of “RMSD pruned”? What do Structure A and Structure B imply in the context of RMSD values presented in the table?
5. Ultimately, authors conclude that Alphafold and/or short MD simulations alone are not apt for predicting structural effects of mutations which is already known in the field. While the authors acknowledge that large-scale simulations could potentially predict the structural effects of mutations, they haven’t cited a recent study (https://doi.org/10.48550/arXiv.2309.03649) which demonstrates that Alphafold combined with enhanced sampling MD simulations could potentially predict the effect of mutations on the structure and dynamics of a protein. Further, the authors mention that the structural predictions by Alphafold are heavily biased by the available structures of Serpin in PDB database. However, they do not discuss the studies demonstrating that this limitation can be circumvented by variations in the input parameters provided for the multiple sequence alignment of Alphafold prediction and combining the structural predictions with the enhanced sampling methods (https://doi.org/10.1016/j.sbi.2023.102645, https://doi.org/10.1021/acs.jctc.2c01189).

   Overall, this study does not provide any new understanding and overlooks existing studies on structural predictions by AlphaFold combined with MD simulations.
