# Supplementary material for: Analysis of AlphaFold and molecular dynamics structure predictions of mutations in serpins
Source: PLoS One. 2024 Jul 5;19(7):e0304451. doi: 10.1371/journal.pone.0304451 (PMC11226102; doi:10.1371/journal.pone.0304451)
Supplement: Attachment — Submitted filename: Response letter R1.pdf [file pone.0304451.s010.pdf]

Dear Dr. Chenette,

Thank you for allowing us to answer the questions arising from our draft manuscript entitled "**Analysis of AlphaFold and molecular dynamics structure predictions of mutations in serpins**", submitted to **PLOS ONE**. We appreciate the time and effort that you and the reviewers have invested in providing us with your feedback. We have discussed all your comments and included changes to reflect the most of your suggestions. We have highlighted the changes in the manuscript.

Additionally, here is a point-by-point response to the reviewers' comments and concerns.

Journal comments

1. *Please ensure that your manuscript meets PLOS ONE's style requirements, including those for file naming. The PLOS ONE style templates can be found at https://journals.plos.org/plosone/s/file?id=wjVg/PLOSOne_formatting_sample_main_body.pdf and https://journals.plos.org/plosone/s/file?id=ba62/PLOSOne_formatting_sample_title_authors_affiliations.pdf*

Thank you for your feedback. We have modified the manuscript format to adapt the document to the requirements. We would like you to confirm whether these changes meet the Journal requirements in the new manuscript version.

2. *We note that the grant information you provided in the 'Funding Information' and 'Financial Disclosure' sections do not match. When you resubmit, please ensure that you provide the correct grant numbers for the awards you received for your study in the 'Funding Information' section.*

We apologize for this mistake. We have tried to modify the "Financial Disclosure" on the submission system. Unfortunately, we were not able to do so. We kindly ask the Editors to consider as correct the stated on the Financial Disclosure in the revised manuscript, and on the Funding Information section of the submission portal.

3. *Your ethics statement should only appear in the Methods section of your manuscript. If your ethics statement is written in any section besides the Methods, please move it to the Methods section and delete it from any other section. Please ensure that your ethics statement is included in your manuscript, as the ethics statement entered into the online submission form will not be published alongside your manuscript.*

We could not find the mentioned ethics statement outside the Methods section in our manuscripts. We would like to kindly ask the Journal if the novel manuscript version is suitable regarding this point.

4. *We note that Figure 1, 2, 3, 4, 5 and 6 in your submission contain copyrighted images. All PLOS content is published under the Creative Commons Attribution License (CC BY 4.0), which means that the manuscript, images, and Supporting Information files will be freely available online, and any third party is permitted to access, download, copy, distribute, and use these materials in any way, even commercially, with proper attribution. For more information, see our copyright guidelines: http://journals.plos.org/plosone/s/licenses-and-copyright. We require you to either (1) present written permission from the copyright*

holder to publish these figures specifically under the CC BY 4.0 license, or (2) remove the figures from your submission:

a. *You may seek permission from the original copyright holder of Figure 1, 2, 3, 4, 5 and 6 to publish the content specifically under the CC BY 4.0 license. We recommend that you contact the original copyright holder with the Content Permission Form (http://journals.plos.org/plosone/s/file?id=7c09/content-permission-form.pdf) and the following text: "I request permission for the open-access journal PLOS ONE to publish XXX under the Creative Commons Attribution License (CCAL) CC BY 4.0 (http://creativecommons.org/licenses/by/4.0/). Please be aware that this license allows unrestricted use and distribution, even commercially, by third parties. Please reply and provide explicit written permission to publish XXX under a CC BY license and complete the attached form." Please upload the completed Content Permission Form or other proof of granted permissions as an ""Other"" file with your submission. In the figure caption of the copyrighted figure, please include the following text: "Reprinted from [ref] under a CC BY license, with permission from [name of publisher], original copyright [original copyright year]."*
b. *If you are unable to obtain permission from the original copyright holder to publish these figures under the CC BY 4.0 license or if the copyright holder's requirements are incompatible with the CC BY 4.0 license, please either i) remove the figure or ii) supply a replacement figure that complies with the CC BY 4.0 license. Please check copyright information on all replacement figures and update the figure caption with source information. If applicable, please specify in the figure caption text when a figure is similar but not identical to the original image and is therefore for illustrative purposes only.*

We find this point may arise from the preprint version of the unreviewed manuscript, available on DOI: https://doi.org/10.1101/2023.01.31.526415. This preprint is authored by us, and we are the copyright owners of these figures. Given the preprint nature of such document, we would like to check if the preprint policy of the Journal is still the detailed in https://journals.plos.org/plosone/s/submission-guidelines#loc-manuscripts-disputing-published-work - Preprints. Nonetheless, following Reviewer 1 advice, we have opted for a major figure redesign, as detailed below herein, in the manuscript and in the submitted figure files. Thus, the figures detailed in this point are no longer present in the manuscript.

Reviewer 1

Review of "Analysis of AlphaFold and molecular dynamics structure predictions of mutations in serpins"

This work seeks to probe whether or not AlphaFold2 (AF2), a structure prediction AI tool, is capable of probing the impact of clinically occurring disease-associated mutations in serpins (serine protease inhibitors). The authors present a biophysical model in which disease-associated mutations cause a conformational change in a model serpin, SERPINC1, and test AF2's ability to predict these changes. Their results demonstrate a key finding that is relevant to a broad audience: that AF2 remains unable to wholly predict global conformational shifts and changes upon mutation/insertion of residues. I laude the authors' clarity in presenting a clear-cut story using a biologically interesting model system that has clinical relevance, and their creative use of drawing upon patient data to select mutations. Not only have the authors presented a clear problem and question, they use as many open-source tools as possible to tackle their hypothesis,

and even shared their data demonstrating a commitment to Open Science. However, at the current stage the manuscript remains largely devoid of necessary details that must be included prior to publication (as described in major revisions). Furthermore, the figures require significant editing to ensure that a reader can understand the data provided. Lastly, while the authors present a very useful biophysical model to probe the power of AF2, they only provide structural details regarding a single state - it will be critical to include structural depictions of both models to allow the reader to compare and contrast any structural predictions.

We thank again Reviewer 1 for their suggestions to improve our manuscript. We have updated such sections in the revised manuscript to try and address the ideas the Reviewer presents on this point.

Major Revisions

1. *The authors do not provide any sort of experimental data in the SI or main text about their immunoelectrophoretic experiments, western blots, PAGE results, and beyond, and should provide the data in the manuscript itself, or provide clear citations to find the relevant data. While the use of this method to study the oligomerization and functional behaviors of their clinical mutants is reasonable, the only data provided is a single table with unitless values, and it remains unclear whether these values were obtained using densitometry or other approaches. I would ask that the authors at least include critically relevant blots that convey the functional impact of their top 5 mutants in the manuscript (or supporting information).*

We apologize for the unclear way to show the methods used in our manuscript. The main objective of this study was to test the accuracy of AlphaFold following MD predictions for mutations with conformational consequences in antithrombin, an anticoagulant serpin. Thus, we considered that a large description of all biochemical methods used to characterize the aberrant forms of antithrombin identified in patients with the selected *SERPINC1* mutations was out of the scope of this manuscript. Moreover, all these methods have been described by our group elsewhere, and all references to these methods are already included in the manuscript and further clarified. Additionally, figures with blots, proteomic or crystallographic data that convey the functional impact of their top 5 mutants in the manuscript generated with carriers of these mutations by our group have been published previously. All these references are included in the manuscript and available for verification.

2. *I request that the authors greatly expand the methods section, both for their experimental work as well as for their computational studies. At present, the details provided on their immunoelectrophoretic methods, separation and unfolding experiments, and semi-quantitative determination are lacking critical details that prevent any reader from trying to replicate their work. These omissions must be fixed prior to publication.*

Again, we apologize for not clearly presenting the references in which experimental data for all variants can be accessed both by reviewers and readers. Following this and the previous points, we have included the references describing these variants in Table 1. We have also included further details on the description of computational methods, adding descriptions and new references for additional details on this matter.

Regarding AlphaFold, we have included additional details: dimerization models were obtained with AlphaFold Multimer. UCSF ChimeraX was used to obtain metrics and figures for AlphaFold

results. Pruned RMSD refers to RMSD computed excluding from the process unstructured or flexible structures to avoid potential biases on the RMSD computations. These results were presented using PyMol. Hydrogen bond analysis was performed with ChimeraX as described elsewhere.

For MD, we create a new section of Materials and Methods, to specify how the original structures were preprocessed to run MD. This section is called "Structure processing". Also, we included in the MD methods the tool used to create the water box (System Builder of Maestro Suite).

3. *While their computational model descriptions are sufficiently detailed for replication, it would be useful if the authors were to expand on the structures, they used to start these simulations, a key missing detail. Additionally, the authors should report the implementation of lDDT and RMSD that was used.*

We appreciate the referee's suggestion of the expansion of the explanation about the method to obtain the structures to run the MD. The crystal structures of Wild Type (1AZX-I) and M5(4EB1-I), and the AlphaFold predictions were used to run the MD. As we said in the revised manuscript, all the structures were preprocessed using the Maestro Tool Protein Preparation Wizard to avoid all problems with hydrogens that could crash between them. The proteins were processed to add the charges using the Maestro tool System Builder.

lDDT:

Regarding the local Distance Difference Test (lDDT) was performed using the lDDT tool of swiss-model (https://swissmodel.expasy.org/lddt/downloads/) , the lDDT score is a measurement of the difference between the local interactions of the two proteins. This tool was used in the CAPS9 (Critical Assessment of protein Structure Prediction 9: https://predictioncenter.org/casp9/index.cgi).

RMSD:

Regarding the Root Mean Square Deviation (RMSD) was calculated using the Maestro tool Simulation Interactions Diagram (SID). This tool takes the first frame as a reference and superimposes all the frames against the reference to evaluate the fluctuation of the protein along the trajectory. The values that are higher than 3 A indicate that the protein is not stable.

The manuscript has been updated to include these explanations.

4. *While the conformational hypothesis the authors present is presented in a logical manner, I would ask that they provide further detail and expand in the introduction on why they think conformation has any basis on the impact of mutations, and that other mechanistic details are not relevant. This detail is needed to demonstrate that the conformational impact of mutations in SERPINC1 are sufficient to predict disease relevance. For instance, if mutations in SERPINC1 instead primarily cause an inability to bind to heparin, resulting in the clinical phenotype, by changing the heparin binding site without any significant conformational changes, prediction of conformational change from the WT state is insufficient to predict clinical relevance. Alternatively, if the primary change is in the likelihood of oligomerization, perhaps a tool like AlphaFold Multimer would be more appropriate (bioRxiv 2021.10.04.463034). The authors current dive into their conformational model*

would be vastly supported by additional explanation as to why conformation dynamics may play a role here.

We acknowledge the correct and appropriate comment of Reviewer 1. The serpin superfamily has a pretty conserved structure needed for them to function, as their native (stressed) conformation allow them to run a suicidal inhibitory mechanism. These mutations disrupt the native (functional) structure, leading either to latent (relaxed) or dimers / polymers, losing the ability to inhibit via the mentioned suicidal mechanism. In the revised manuscript we have expanded the Introduction to support the functional or conformational consequences of mutations in antithrombin and their clinical effects.

Additionally, we have run AlphaFold Multimer to test variants causing dimerization of antithrombin. We found both M2 and M4 do form dimers. Nonetheless, we also find AlphaFold Multimer output dimers for wild-type. Indeed, the dimer structure AlphaFold computes form M2 and M4 is the same as the computed for the wild-type sequence, exchanging one β-strand with the A sheet of the other molecule. Moreover, the dimer computed does not correspond to the experimentally determined models (such as PDB: 2ZNH, Fig 1D of revised manuscript), in which two β-strands are exchanged between the two counterparts. We include images presenting each scenario (S2 Fig. of revised manuscript) for further clarification on our results regarding AlphaFold Multimer, highlighting in purple the β-strands exchanged.

5. *Importantly, I would ask that the authors provide *both* structural models of their two conformations ("stressed", represented by 1AZX in the PDB, and "relaxed", represented by 4EB1) in their figures and explicitly clarify that the "p.Glu241…" variant was crystalized by the same lab (PDB: 4EB1). At present, it seems like the authors are only presenting figures of the "stressed" conformation and not providing any further figures/structural details on the disease-associated relaxed state, despite it being more predictive of disease-association. It would be important to provide the reader with structural details of both states, and to overlay them alongside the AF2 predictions, to allow for visual comparison. In particular, after examining the 4EB1 and 1AZX structures, it is not immediately obvious what the consequences of the additional strand in the beta-sheet are. Most of the structure is the same, with an RMSD between the chain "I" of both structures ~1.268 Å according to PyMol. I can imagine that there could be significant changes, but making them clearer in the paper is essential.*

Again, we would like to thank Reviewer 1 on their comments, as we think their suggestions allowed us to greatly improve our manuscript. Following their advice, we've stated that 4EB1 was obtained by our group in the revised manuscript.

Regarding the relaxed state, we have included a figure presenting this conformation (Fig 1C in the revised manuscript, 1AZX-L) to clarify the differences with the stressed structure (Fig 1A, 1AZX-I). Furthermore, we also included figures comparing 1AZX-I (native) and 4EB1 (M5, hyperstable) crystals with AlphaFold predictions, on Fig 4A and Fig 5 on the revised manuscript, respectively.

Finally, relaxed structure means to lose the active center of the protein, thus it is rendered inactive, leading this to pathology. We have updated the manuscript, as stated above, to try and further clarify the mechanism linking conformational changes of serpins with pathology.

6. *It remains unclear why Molecular dynamics (MD) was used in this study, particularly relative to the use of AF2. It would be helpful for the authors to clarify in the introduction why they chose to also study MD simulation-based conformational shifts alongside AF2, and why equilibrium MD was chosen over other methods that have been shown to predictively study the impact of mutations (such as free energy calculations). Otherwise, the focus of this paper should be entirely on AF2 and the MD work can be disregarded entirely.*

We appreciate the referee's inquiry regarding the use of Molecular Dynamics (MD) simulations alongside AlphaFold2 (AF2) predictions in our study. We realize our initial explanation might not have fully articulated the scientific reasoning behind this approach.

MD was employed as a complementary tool to AF2 predictions. While AF2 provided us with the predicted native structures for all antithrombin inputs, MD simulations allowed us to explore the dynamic behavior of these proteins over time. Specifically, we were interested in whether the AF2-predicted structures would exhibit any propensity to transition towards relaxed structures under dynamic conditions. This aspect of protein behavior, which involves the exploration of conformational landscapes over time, is something that static structural predictions from AF2 alone cannot capture.

The decision to use equilibrium MD over other methods, such as free energy calculations, was based on our specific research objectives. Equilibrium MD simulations offer a way to observe the spontaneous conformational changes of proteins in a simulated physiological environment, thereby providing insights into the potential flexibility and stability of the predicted structures. In contrast, methods like free energy calculations, while valuable for understanding the thermodynamics of specific conformational states or mutations, do not inherently provide the temporal resolution or the broader exploration of the conformational space that equilibrium MD does.

Thus, the integration of MD with AF2 predictions was crucial in our study for a more comprehensive understanding of the structural dynamics of antithrombin. The MD simulations complemented the AF2 predictions by providing a dynamic perspective, which is essential for understanding the full spectrum of protein behavior, especially in the context of potential pathogenic mutations.

We hope this clarification underscores the rationale behind our methodological choices and the value they add to our study. We believe that the combined use of AF2 and MD offers a more holistic understanding of protein behavior, which is vital for our research objectives.

7. *The authors describe doing 1 µs (1000 ns) of MD simulation to study the impact of mutations, but do not provide any evidence that this simulation time is sufficient sampling to study the impact of mutations. Is there kinetic evidence that the conformational relaxation (or transition from stressed to relaxed) occurs in the timeframe of 1000 ns? Providing precedent and/or kinetic information as to why the amount of simulation time was chosen will be critical. Alongside this, it would be good to show some form of simulation convergence (even in the SI) to demonstrate that they have sufficiently sampled the reasonable conformations of SERPINC1.*

We thank the referee for bringing up an important aspect of our MD simulation methodology. We acknowledge that providing a clear rationale for the simulation time and demonstrating the sufficiency of our sampling is crucial for the credibility of our results.

In our original manuscript, we mentioned conducting 1 µs (1000 ns) MD simulations to study the impact of mutations on *SERPINC1*. This duration was chosen based on a balance between computational feasibility and the need to allow for significant conformational changes. While it is true that specific kinetic data on serpin conformational transitions is lacking, our decision was informed by a review of the literature on similar protein systems. In several studies involving comparable protein sizes and complexities, simulation times ranging from several hundred nanoseconds to a few microseconds have been shown to capture essential conformational changes.

To further validate our simulation time, we conducted additional simulations that were extended to 1 µs. This tenfold increase in duration was intended to provide a more robust test for conformational stability and transitions. However, we acknowledge that even this extended time may not fully capture all possible conformational states, given the complexity and potential slow kinetics of serpin conformational changes.

Regarding temperature variations, we explored simulations at 40ºC to mimic fever conditions and at 100ºC as an extreme case to induce potential structural transitions. While these conditions are non-physiological, they were intended to stress-test the stability of the predicted structures under various thermal conditions.

We hope these additional details and analyses provide the necessary assurance that our simulation time and conditions were thoughtfully chosen and that our MD simulations offer valid insights into the impact of mutations on *SERPINC1*.

8. *The figures require extensive clarification and reworking, as described below:*
    a. *Each figure appears to be pixelated images upon printing, which makes it incredibly difficult to interpret the structures provided in the figures. Higher resolution structural renderings are required.*

We thank again Reviewer 1 for their comments. We have redesigned the manuscript figures, merging some, removing uninformative others, and creating pictures with higher quality.

b. *Even with the figure captions it remains unclear what takeaways one should have from the structural representations, and what the structures provided even represent. I ask that the authors greatly expand in the figure captions what the major takeaways of their data are and include legends in the figures themselves to help the reader understand what they are looking at.*

Following Reviewer 1 suggestions, we have tried to clarify the take-home message on each figure of the revised manuscripts. Moreover, we have modified the figures to try and better synthesize the messages arising from our work.

c. *Further analytical methods for each figure (i.e., referencing the relevant figure in the methods section) are required to ensure that a reader can reproduce the results of this work. I ask that the authors provide further analytical details as to how some of their figure computations were done.*

We want to acknowledge the suggestion of the reviewer to provide details of the analysis. To clarify this point, we add in the metrics the specifications of the analysis. In this case, to evaluate the changes produced in the secondary structure of the proteins along the trajectory, the Maestro Simulation Interactions Diagram (SID) tool was used to show the evolution of the alpha helices and beta-strands along the whole simulation. This tool analyzes many properties

of the protein such as RMSD, RMSF, and SSE (Secondary Structure Elements). SID shows a summary of the SSE in all residues of the protein, so SSE analysis can be used to evaluate if a residue (or a group of residues) changes its secondary structure along the simulation.

Also, Figure 6 of the revised manuscript was obtained using the Maestro Simulation Interactions Diagram (SID) tool under version 2020-04.

d. *In Figure 7, the authors try to demonstrate the hydrogen-bonding network shifts between structures. However, the format they present the comparison of hydrogen bonding networks is difficult to interpret due to the nature of the venn diagrams used. I would suggest that authors refer to some previous efforts to map out hydrogen bonding networks and try to mimic those kinds of visual representations. A potentially useful reference point can be found in: Oh, S., Paknejad, N. & Hite, R. K. Gating and selectivity mechanisms for the lysosomal K+channel TMEM175. eLife doi:10.7554/eLife.53430 Comprehensive information about all changes in the hydrogen bonding is much less useful as a main text figure than some specific analyses of relevant changes in the networks. See below for some more suggestions of relevant analyses.*

We want to acknowledge the comment of the reviewer regarding Figure 7. Due to the difficulty of concluding with Venn's Diagram, to facilitate the understanding of the differences between each hydrogen-bonding network we put a table (Table 5) in the revised manuscript instead of Figure 7. With this table, we show how there are more similarities in the hydrogen-bonding network between the prediction of the AlphaFold and M1-M3 than the crystal of wild-type (1AZX-I) with the prediction of AlphaFold.

9. *The authors provide an RMSD overlay to demonstrate where "differences" occur between structural predictions from AF2 and the crystal structure - I ask that the authors expand on this to provide needed details on how these overlays were generated and RMSDs computed. At present it remains unclear what atoms were used to compute the RMSD, what regions were studied for large changes in RMSD, and what a "cutoff" was for choosing which regions were "different" enough, and how that cutoff would have been chosen. If the differences were chosen visually, it would be important for the authors to make that explicitly clear and provide precedent as to why visual inspection is sufficient for studying structural changes in SERPINC1. Alternatively, it is common to color the cartoon of a structure by its RMSD to another state (usually using the B-factor field in a PDB file), which provides a more comprehensive picture of structural changes). Lastly, calculating RMSD to wild type is much less useful than calculating it to both wild type and disease-relevant states. In the prospective case, one could imagine using AF2 to predict the structure of the variant, and if the structure has a lower RMSD to the disease-relevant state, this would suggest that this is a disease-relevant variant. It would be more useful to compare RMSD to the disease-relevant state to assess similarity to the disease-induced conformation than WT.*

We appreciate the reviewer's detailed feedback regarding our RMSD overlay analysis between AlphaFold2 (AF2) predictions and the crystal structure. In response to the concerns raised, we have expanded the explanation in the Metrics section of the Materials and Methods part of our manuscript.

10. *The authors should greatly expand their conclusion section. While the statement "the old protein folding problem remains, in some respects, unsolved" is supported by the data presented here, it is far from a good use of the conclusion section to make such grandiose statements and not provide further insight, discussion, or summary into the work done here. It would be useful for the authors to provide further summary of their work, discuss any caveats to be aware of, and possible future directions this work could be taken in.*

We thank Reviewer 1 again for their suggestions to improve our manuscript. We have updated such sections in the revised manuscript to try and adress the ideas the Reviewer presents in this point.

Minor Revisions

1. *It would be useful for the authors to expand the introduction to and discussions to further discuss and clarify their hypothesis for this research work, as well as include a discussion on whether AF2 could be used prospectively given their results.*

In the updated manuscript, we have extended the conclusions section, as well as included insights in the Introduction and Discussion. In these changes, we now state the need to enhance current tools (including AF in those) to accurately predict mutant proteins.

2. *It would be useful for the authors to cite other efforts of studying AF2 and its ability to probe the impact of mutations on conformation. Particularly, it would be good to cite papers that have also built on this previous work and frame the present work in the current context (PLOS ONE 18, e0282689 (2023))*

We acknowledge the efforts made by other authors in exploring the capabilities of AlphaFold2 (AF2) to assess the impact of mutations on protein conformation. As suggested, we have ensured that relevant literature, including the study mentioned by the referee (PLOS ONE 18, e0282689 (2023)), is appropriately cited in our manuscript. This reference is indeed included in our manuscript under number [20], both in the previous and updated versions.

Building on the premise of the cited study, which focuses on testing AlphaFold with missense variants, we sought to broaden the scope of research in this area. Our study extends the examination of AF capabilities to include more complex variants, observed in real-life patients. This approach allows us to provide a deeper and more comprehensive understanding of the predictive power of AF2, particularly in the context of clinically relevant and structurally significant mutations.

By exploring these more intricate variants, we aim to contribute a novel perspective to the ongoing discourse on the utility and limitations of computational tools in predicting protein structural alterations due to mutations.

3. *The phrase "time-dependent processes cannot be parallelized" is in general false and should be removed when discussing MD. This is the basis for the work done with Folding@home and many other MD methods (Examples include: Nat. Chem. 13, 651–659 (2021)., Annu Rev Biophys. 2017 May 22;46:43-57.). It would be good for the authors to appropriately reference the challenges with modeling time-dependent processes, even with parallelization.*

We appreciate the referee's comment regarding our statement on the parallelization of time-dependent processes in Molecular Dynamics (MD) simulations. We agree that our wording

was overly broad and potentially misleading, as it overlooked the significant advancements in the field of MD simulations, particularly in terms of parallelization.

In light of this, we have revised the manuscript to more accurately reflect the current capabilities and limitations of parallelization in MD. The revised text now reads: "While parallelization techniques have significantly advanced the field of MD simulations, allowing for more complex and longer simulations (https://www.nature.com/articles/s41557-021-00707-0), there remain inherent challenges in modeling time-dependent processes even with parallelization."

This revision not only corrects the inaccuracies in our original statement but also provides a more comprehensive understanding of the complexities associated with simulating time-dependent processes in MD, even when leveraging parallel computing techniques.

We hope that these changes address the referee's concerns and enhance the accuracy and quality of our manuscript

4. *It would improve readability to replace the long variant names with shortened names (and potentially list these in a table).*

We are aware that the names of the mutations selected on this manuscript may result in a complicated lecture of the manuscript. We have created shorter IDs for each mutations following the Reviewer's advice.

5. *For the figure of the 4EB1 variant, the figure would be better served by rotating the protein such that the beta sheet containing the major change is facing the reader directly.*

We have updated the figures on the revised manuscript. Now, on Fig 5 of the updated version the sheet A is presented in a clearer way for both 4EB1 and AF prediction for M5.

Reviewer 2

In this manuscript, authors have used Alphafold, Rosetta and short molecular dynamics simulations to predict structural properties of 5 mutants/variants of serpins. As it is already known in thefield, the authors find that these tools are not powerful enough to predict the effect sof mutations on the protein structure. Thus, this study does not provide any new insights. Besides, the authors have not provided sufficient details on the methods and analysis presented in this manuscript. Therefore, I cannot recommend this manuscript for publication. Please find my specific comments below:

We sincerely thank Reviewer 2 for their valuable feedback and constructive criticism. We have taken this opportunity to refine our manuscript and address the concerns raised.

<u>Regarding the Novelty and Insight of Our Study:</u>

While we recognize that the limitations of tools like AlphaFold (AF), Rosetta, and MD simulations in predicting the effects of mutations on protein structure are known, our study explores these tools in the context of complex and clinically relevant mutations in serpins. Our work extends beyond routine mutation analysis by examining not only single amino acid substitutions but also more complex variants such as a 10 amino acid insertion. This insertion

represents a significant structural alteration, offering a novel case study to test the predictive capabilities of these tools.

Our findings indeed confirm the limitations of current computational tools in accurately predicting structural changes due to mutations. However, the value of our study lies in its exploration of these limitations in the context of complex mutations, which are less studied but highly relevant in clinical settings. The insights gained from our analysis contribute to a deeper understanding of where AlphaFold and similar tools could be improved, particularly regarding their dependence on PDB structures and lack of physicochemical considerations in mutation predictions.

This aspect of our research is not just a repetition of known limitations but an exploration of how these tools perform under more challenging conditions, providing valuable insights for future enhancements and applications in protein structural biology.

Regarding Methodological Details:

We understand the reviewer's concern about insufficient methodological details. In response, we have thoroughly revised the Methods section of our manuscript to provide a more comprehensive and detailed explanation of our computational approaches. This includes specifics on how we used AlphaFold, Rosetta, and MD simulations. We have also expanded our analysis section to clearly articulate how we interpreted the results from these simulations and their implications for understanding the structural impact of mutations in serpins.

We believe these revisions significantly enhance the clarity and depth of our manuscript, addressing the concerns raised by the reviewer.

Furthermore, as noted, our study aligns with the philosophy of previous research published in this journal by Pak et al. (DOI: 10.1371/journal.pone.0282689), indicating a recognized interest in this area of research. While the cited study focused on single amino acid changes, our work expands this approach to more complex variants, thereby broadening the scope and relevance of computational predictions in the context of real-life clinical scenarios.

We hope these clarifications and enhancements address the concerns raised by Reviewer 2 and demonstrate the unique contributions of our study to the field.

1. *Authors describe various conformational states of serpins using the terms "native", "stressed", "hyperstable", and "relaxed structure". However, these terms are not properly defined in the introduction and there is an ambiguity in the usage of these terms in the manuscript. This makes it difficult to understand which structural state is being discussed. Authors must define these terms and be consistent in the usage of these terms throughout the manuscript. Besides, figures showing these structural states is required for the purpose of clarity.*

Following Reviewer 2 comments, we have updated the Introduction to better explain these terms for readers not familiar with the serpins structural conformations. Furthermore, new figures have been included in the manuscript presenting both native and latent structures (Fig 1A and 1C in the revised manuscript).

2. *2.In the methods section, it is mentioned that the data was collected from 350 patients, and 135 unique genetic variations were found among 250 samples. What about*

remaining 100 patients? Further, the authors mention that anti-FXa and anti-FIIa activities measured. Authors must explain anti-FXa and anti-FIIa activities.

We would like to further clarify this point mentioned by Reviewer 2. 100 patients with antithrombin deficiency had no *SERPINC1* variants. Some of these patients had transient or acquired antithrombin deficiency, and others had different mechanisms explaining antithrombin deficiency such as glycosylation disorders as we also described before (DOI: 10.3324/haematol.2009.015487). To better described the sample of patients we studied, source of the mutations included in this study, we have included a new figure (Figure 2 in the updated manuscript) showing the flow chart of patients evaluated. We have also explained the meaning of evaluating anti-FXa and anti-FIIa activities in patients with antithrombin deficiency.

3. *What was the reference structure used for computing RMSD values given in Table 2?*

We acknowledge the reviewer's comment about the reference structure. The reference structures of the wild-type and M5 are obtained from the AlphaFold predictions using the full database, and each crystal structure was compared against its prediction of AlphaFold using the small database. Table 2 shows the absence of differences between the predictions using both databases.

4. *In Table 3, what is the meaning of "RMSD pruned"? What do Structure A and Structure B implyin the context of RMSD values presented in the table?*

We acknowledge the reviewer's question about the meaning of "RMSD pruned". This nomenclature is used by the software ChimeraX, which is part of the analysis. ChimeraX computes RMSD pruned excluding the regions with more flexibility of the protein. With this approximation, the value of RMSD is more refined and the provided information is more accurate than the classical RMSD value.

Regarding structure A, we selected the AlphaFold prediction of the wild-type to compare against the crystal and the AlphaFold predictions of the mutants. So, we want to compare the prediction of the Wild Type against the real state of the protein (1AZX) and the other predictions.

Regarding structure B, we selected the crystal structure of the M5 to compare against the AlphaFold predictions of the M5 and wild-type. So, we want to compare the prediction of the real state of the M5 protein (4EB1), regarding the M5 and the wild-type predictions.

5. *Ultimately, authors conclude that Alphafold and/or short MD simulations alone are not apt for predicting structural effects of mutations which is already known in the field. While the authors acknowledge that large-scale simulations could potentially predict the structural effects of mutations, they haven't cited a recent study (https://doi.org/10.48550/arXiv.2309.03649) which demonstrates that Alphafold combined with enhanced sampling MD simulations could potentially predict the effect of mutations on the structure and dynamics of a protein. Further, the authors mention that the structural predictions by Alphafold are heavily biased by the available structures of Serpin in PDB database. However, they do not discuss the studies demonstrating that this limitation can be circumvented by variations in the input parameters provided for the multiple sequence alignment of Alphafold prediction and combining the structural predictions with the enhanced sampling*

methods(https://doi.org/10.1016/j.sbi.2023.102645, https://doi.org/10.1021/acs.jctc.2c01189). Overall, this study does not provide any new understanding and overlooks existing studies on structural predictions by AlphaFold combined with MD simulations.

Again, we understand the Reviewer 2 concerns, and we thank the opportunity to address them.

Regarding the mentioned study by Vani et al. (DOI: 10.48550/arXiv.2309.03649), they use their own AlphaFold2 based approach AlphaFold2-RAVE), to provide conformational ensembles for biomolecules. In our case, we see antithrombin structures predicted by AlphaFold are pretty stable, even when we introduce different sequences, as M4, or the more remarkable M5 complex insertion. Moreover, in the mentioned study authors work with proteins "extremely sensitive to small changes in sequence", such as kinases. It is not the case of antithrombin, whose mutations could lead to different type I / II deficiencies, including not just structural consequences, but also disruption of electrostatic interactions. In fact, p.R79C does not lead to a conformational change, but to an impairment on the antithrombin interaction with heparin. Instead, we find antithrombin is more sensitive to environmental changes, such as temperature, which as mentioned above, is not currently modeled on AF simulations, and could be a possible enhancement for future AF implementations.

Regarding AF suggested biases, Reviewer 2 references two studies stating how to enhance sampling. In this case, we do not only work with natural antithrombin structures, but with aberrant conformations, a different scenario as presented in such studies.

Nonetheless, reading these works, we find that Meller at al. (DOI:10.1021/acs.jctc.2c01189) looked for possible open pockets, based on the hypothesis that AF may capture small openings on such pockets. To try and maximize those, they change MSA parameters, and then run MD simulations at 100 ns (like we do in our study for MD). The problem we find is that AF does not show the slightest change in its predictions for these mutations. Thus, as this approach is based on maximizing possible changes, as we see none, we conclude that this approach (AF) would not improve our results. Moreover, the changes seen in this article are restricted to small changes on specific parts of the protein, whilst the conformations experimentally verified for the mutations selected in our study mean a bigger rearrangement of the protein structure compared to the changes observed in the mentioned study.
