# Supplementary material for: Analysis of AlphaFold and molecular dynamics structure predictions of mutations in serpins
Source: PLoS One. 2024 Jul 5;19(7):e0304451. doi: 10.1371/journal.pone.0304451 (PMC11226102; doi:10.1371/journal.pone.0304451)
Supplement: Attachment — Submitted filename: Response letter R2.pdf [file pone.0304451.s011.pdf]

R2: response letter

PONE-D-23-28110R1

Analysis of AlphaFold and molecular dynamics structure predictions of mutations in serpins

Dear Dr. Chenette,
Thank you for allowing us to answer the questions arising from our draft manuscript, "Analysis of AlphaFold and molecular dynamics structure predictions of mutations in serpins, " submitted to PLOS ONE. We appreciate the time and effort that you and the reviewers have invested in providing us with your feedback. We have discussed all your comments and included changes to reflect most of your suggestions. We have highlighted the changes in the manuscript. Here is a point-by-point response to the reviewers' comments and concerns.

Reviewer 3

1. The authors have used no template and BDF for the structure prediction via AF. But what happens when we use wild type as a template to predict the mutant state? Does it yield a better structure prediction?

First, we thank Reviewer 3 for their comments on how to improve our manuscript.

AlphaFold relies on a custom database designed by DeepMind called Best Fantastic Database (BFD). AlphaFold developers allow users to choose between running the model relying on a full BFD (containing most sequences and structures logged in public databases) or a reduced version of the BFD. Thus, the capabilities of AlphaFold entirely rely on this BFD. In both cases, multiple proteins can affect the final result.

AlphaFold users cannot manually select a single input for the model to produce a result. Moreover, in that scenario, the output of AlphaFold may not be comparable to the results produced by both complete or reduced AlphaFold for the reason above.

Nonetheless, we have tested results comparing complete and reduced AlphaFold for wild-type and M5 sequences, finding no significant differences between the two results for any of the sequences tested. These results are presented in Table 2 of the manuscript.

2. As we know the basics of any structure prediction is comparing the dihedrals (chi, phi, psi, and omega) of mutant residue predicted with PDB structure. Computing RMSD and RMSFs does not zoom into the effect of mutations. This can be easily done for M5.

We are grateful to the reviewer for their suggestion. We calculated the dihedral angles of the residues near mutation (S204, E205, A206, I207, N208, T211, V212, L213, V214, and L215). When we compared the three previous residues after the mutation, we found differences between the crystal structure of the M5 and the prediction of AlphaFold. The crystal structure has β-sheets before the mutation, whilst the

AlphaFold prediction introduces a right-handed α-helix. These results agree with those presented in Tables 5 and 6 and page 16 lines 319-323 (manuscript with tracking).

We also updated the manuscript with tables (S6 Table and S7 Table) containing information about dihedral angles.

3. Is there experimental evidence that validates the REU analysis in Table 4 that all mutations have a destabilizing effect?

This is an interesting point. As the mutations selected have been studied previously using experimental techniques, we have information on their behavior, mechanisms leading to disease, and other metrics.

For M1, the mechanism is on residue 79 itself. As Arg79 is a critical residue that directly interacts with heparin, the mutation to Cys (losing the positive charge associated with the Arg) severely impairs the interaction between antithrombin and heparin. The differential interaction between the WT molecule and the M1 variant with heparin was proved experimentally by crossed immunoelectrophoresis in the presence of heparin. Figure A shows the differential migration pattern of the molecules bound or not bound to heparin, both in the plasma of a healthy subject and in plasma of a heterozygous carrier of the p.Arg79Cys variant, as well as in recombinant WT and M1 variants. Thus, the pathological mechanism of this mutation is not structure-based, so we would not expect structure disruptions for this mutation.

Figure A. Electrophoretic assay of WT (Arg79) and M1 (Cys79) antithrombins by their interaction with heparin. Differences of plasma and recombinant antithrombins are shown.

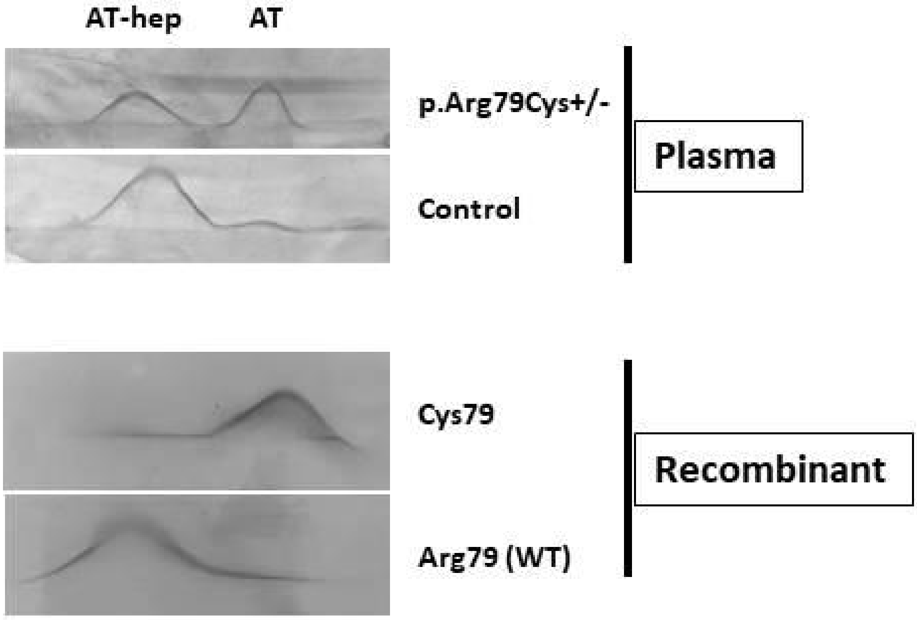

The mechanism involved in antithrombin deficiency is different for mutations M2 and M3. M2 (p.Pro112Ser) leads to deficiency by creating abnormal antithrombin polymers that are mainly retained inside the cells. However, traces of disulphide-linked mutant dimers are detected in plasma, as our group has published elsewhere (J. Corral *et al.*, “Mutations in the shutter region of antithrombin result in the formation of disulfide-linked dimers and severe venous thrombosis,” *J. Thromb. Haemost.*, vol. 2, no. 6, pp. 931–939, 2004, doi: 10.1111/j.1538-7836.2004.00749.x). In this case, the mutation creates a structural disruption, and it is in line with the REU result for M2.

The last missense mutation, M3 (p.Met283Val), leads antithrombin to fold into a latent confirmation, with the RCL inserted as a new beta-strand in sheet A. The variant is secreted but has no anticoagulant activity and low affinity for heparin, as our group demonstrated previously (M. de la Morena-Barrio *et al.*, “High levels of latent antithrombin in plasma from patients with antithrombin deficiency,” *Thromb. Haemost.*, vol. 117, no. 5, pp. 880–888, 2017, doi: 10.1160/TH16-11-0866). There is a structural disruption in this case, but it is not as detected as in the M2 case with the REU metrics.

Thus, we find higher values of REU (more destabilization) for one of the mutations whose mechanism is based on structural disruptions (M2). Still, in contrast, we got lower REU values for M3 (structural disruption) than for M1 (whose mechanism does not change the protein structure). Overall, we find ROSIE 2 predictions may help to

determine the pathogenicity potential of some mutations, but there seem to be better methods to evaluate missense mutations in serpins altogether.

We have included these observations in the new version of the manuscript to further clarify ROSIE 2's potential for assessing the pathogenicity of missense variants in serpins.

4. In Table 2 RMSD was done for which atoms?

Table 2 was computed using the heavy atoms of each structure pair, as provided by PyMol RMSD calculations.

We would like to thank Reviewer 3 again for their feedback. Their suggestions have improved the quality of the manuscript.
