# Supplementary material for: Analysis of AlphaFold and molecular dynamics structure predictions of mutations in serpins
Source: PLoS One. 2024 Jul 5;19(7):e0304451. doi: 10.1371/journal.pone.0304451 (PMC11226102; doi:10.1371/journal.pone.0304451)
Supplement: Attachment — Submitted filename: 2023-Oct-12-PLOSOne-Serpins-Review.docx [file pone.0304451.s008.docx]

**Review of “Analysis of AlphaFold and molecular dynamics structure predictions of mutations in serpins”**

This work seeks to probe whether or not AlphaFold2 (AF2), a structure prediction AI tool, is capable of probing the impact of clinically occurring disease-associated mutations in serpins (serine protease inhibitors). The authors present a biophysical model in which disease-associated mutations cause a conformational change in a model serpin, SERPINC1, and test AF2’s ability to predict these changes. Their results demonstrate a key finding that is relevant to a broad audience: that AF2 remains unable to wholly predict global conformational shifts and changes upon mutation/insertion of residues. I laude the authors’ clarity in presenting a clear-cut story using a biologically interesting model system that has clinical relevance, and their creative use of drawing upon patient data to select mutations. Not only have the authors presented a clear problem and question, they use as many open-source tools as possible to tackle their hypothesis, and even shared their data demonstrating a commitment to Open Science. However, at the current stage the manuscript remains largely devoid of necessary details that must be included prior to publication (as described in major revisions). Furthermore, the figures require significant editing to ensure that a reader can understand the data provided. Lastly, while the authors present a very useful biophysical model to probe the power of AF2, they only provide structural details regarding a single state - it will be critical to include structural depictions of both models to allow the reader to compare and contrast any structural predictions.

**Major Revisions:**

1. The authors do not provide any sort of experimental data in the SI or main text about their immunoelectrophoretic experiments, western blots, PAGE results, and beyond, and should provide the data in the manuscript itself, or provide clear citations to find the relevant data. While the use of this method to study the oligomerization and functional behaviors of their clinical mutants is reasonable, the only data provided is a single table with unitless values, and it remains unclear whether these values were obtained using densitometry or other approaches. I would ask that the authors at least include critically relevant blots that convey the functional impact of their top 5 mutants in the manuscript (or supporting information).
2. I request that the authors greatly expand the methods section, both for their experimental work as well as for their computational studies. At present, the details provided on their immunoelectrophoretic methods, separation and unfolding experiments, and semi-quantitative determination are lacking critical details that prevent any reader from trying to replicate their work. These omissions must be fixed prior to publication.
3. While their computational model descriptions are sufficiently detailed for replication, it would be useful if the authors were to expand on the structures, they used to start these simulations, a key missing detail. Additionally, the authors should report the implementation of lDDT and RMSD that was used.
4. While the conformational hypothesis the authors present is presented in a logical manner, I would ask that they provide further detail and expand in the introduction on why they think conformation has any basis on the impact of mutations, and that other mechanistic details are not relevant. This detail is needed to demonstrate that the conformational impact of mutations in SERPINC1 are sufficient to predict disease relevance. For instance, if mutations in SERPINC1 instead primarily cause an inability to bind to heparin, resulting in the clinical phenotype, by changing the heparin binding site without any significant conformational changes, prediction of conformational change from the WT state is insufficient to predict clinical relevance. Alternatively, if the primary change is in the likelihood of oligomerization, perhaps a tool like AlphaFold Multimer would be more appropriate (bioRxiv 2021.10.04.463034). The authors current dive into their conformational model would be vastly supported by additional explanation as to why conformation dynamics may play a role here.
5. Importantly, I would ask that the authors provide *both* structural models of their two conformations (“stressed”, represented by 1AZX in the PDB, and “relaxed”, represented by 4EB1) in their figures and explicitly clarify that the “p.Glu241…” variant was crystalized by the same lab (PDB: 4EB1). At present, it seems like the authors are only presenting figures of the “stressed” conformation and not providing any further figures/structural details on the disease-associated relaxed state, despite it being more predictive of disease-association. It would be important to provide the reader with structural details of both states, and to overlay them alongside the AF2 predictions, to allow for visual comparison. In particular, after examining the 4EB1 and 1AZX structures, it is not immediately obvious what the consequences of the additional strand in the beta-sheet are. Most of the structure is the same, with an RMSD between the chain “I” of both structures ~1.268 Å according to PyMol. I can imagine that there could be significant changes, but making them clearer in the paper is essential.
6. It remains unclear why Molecular dynamics (MD) was used in this study, particularly relative to the use of AF2. It would be helpful for the authors to clarify in the introduction why they chose to also study MD simulation-based conformational shifts alongside AF2, and why equilibrium MD was chosen over other methods that have been shown to predictively study the impact of mutations (such as free energy calculations). Otherwise, the focus of this paper should be entirely on AF2 and the MD work can be disregarded entirely.
7. The authors describe doing 1 µs (1000 ns) of MD simulation to study the impact of mutations, but do not provide any evidence that this simulation time is sufficient sampling to study the impact of mutations. Is there kinetic evidence that the conformational relaxation (or transition from stressed to relaxed) occurs in the timeframe of 1000 ns? Providing precedent and/or kinetic information as to why the amount of simulation time was chosen will be critical. Alongside this, it would be good to show some form of simulation convergence (even in the SI) to demonstrate that they have sufficiently sampled the reasonable conformations of SERPINC1.
8. The figures require extensive clarification and reworking, as described below:
   a. Each figure appears to be pixelated images upon printing, which makes it incredibly difficult to interpret the structures provided in the figures. Higher resolution structural renderings are required.
   b. Even with the figure captions it remains unclear what takeaways one should have from the structural representations, and what the structures provided even represent. I ask that the authors greatly expand in the figure captions what the major takeaways of their data are and include legends in the figures themselves to help the reader understand what they are looking at.
   c. Further analytical methods for each figure (i.e., referencing the relevant figure in the methods section) are required to ensure that a reader can reproduce the results of this work. I ask that the authors provide further analytical details as to how some of their figure computations were done.
   d. In Figure 7, the authors try to demonstrate the hydrogen-bonding network shifts between structures. However, the format they present the comparison of hydrogen bonding networks is difficult to interpret due to the nature of the venn diagrams used. I would suggest that authors refer to some previous efforts to map out hydrogen bonding networks and try to mimic those kinds of visual representations. A potentially useful reference point can be found in: Oh, S., Paknejad, N. & Hite, R. K. Gating and selectivity mechanisms for the lysosomal K+channel TMEM175. eLife doi:10.7554/eLife.53430

Comprehensive information about all changes in the hydrogen bonding is much less useful as a main text figure than some specific analyses of relevant changes in the networks. See below for some more suggestions of relevant analyses.

9. The authors provide an RMSD overlay to demonstrate where “differences” occur between structural predictions from AF2 and the crystal structure - I ask that the authors expand on this to provide needed details on how these overlays were generated and RMSDs computed. At present it remains unclear what atoms were used to compute the RMSD, what regions were studied for large changes in RMSD, and what a “cutoff” was for choosing which regions were “different” enough, and how that cutoff would have been chosen. If the differences were chosen visually, it would be important for the authors to make that explicitly clear and provide precedent as to why visual inspection is sufficient for studying structural changes in SERPINC1.

Alternatively, it is common to color the cartoon of a structure by its RMSD to another state (usually using the B-factor field in a PDB file), which provides a more comprehensive picture of structural changes). Lastly, calculating RMSD to wild type is much less useful than calculating it to both wild type and disease-relevant states. In the prospective case, one could imagine using AF2 to predict the structure of the variant, and if the structure has a lower RMSD to the disease-relevant state, this would suggest that this is a disease-relevant variant. It would be more useful to compare RMSD to the disease-relevant state to assess similarity to the disease-induced conformation than WT.

10. The authors should greatly expand their conclusion section. While the statement “the old protein folding problem remains, in some respects, unsolved” is supported by the data presented here, it is far from a good use of the conclusion section to make such grandiose statements and not provide further insight, discussion, or summary into the work done here. It would be useful for the authors to provide further summary of their work, discuss any caveats to be aware of, and possible future directions this work could be taken in.

**Minor Revisions**

1. It would be useful for the authors to expand the introduction to and discussions to further discuss and clarify their hypothesis for this research work, as well as include a discussion on whether AF2 could be used prospectively given their results.
2. It would be useful for the authors to cite other efforts of studying AF2 and its ability to probe the impact of mutations on conformation. Particularly, it would be good to cite papers that have also built on this previous work and frame the present work in the current context (PLOS ONE 18, e0282689 (2023) )
3. The phrase “time-dependent processes cannot be parallelized” is in general false and should be removed when discussing MD. This is the basis for the work done with Folding@home and many other MD methods (Examples include: Nat. Chem. 13, 651–659 (2021)., Annu Rev Biophys. 2017 May 22;46:43-57.). It would be good for the authors to appropriately reference the challenges with modeling time-dependent processes, even with parallelization.
4. It would improve readability to replace the long variant names with shortened names (and potentially list these in a table).
5. For the figure of the 4EB1 variant, the figure would be better served by rotating the protein such that the beta sheet containing the major change is facing the reader directly.
